# 1-(4-Chloro­phen­yl)-3-(2,4-dichloro­benzo­yl)thio­urea

**DOI:** 10.1107/S160053680900004X

**Published:** 2009-01-08

**Authors:** M. Khawar Rauf, Michael Bolte, Amin Badshah

**Affiliations:** a Department of Chemistry, Quaid-i-Azam University, Islamabad 45320, Pakistan; b Institut für Anorganische Chemie, J.-W.-Goethe-Universität Frankfurt, Max-von-Laue-Strasse 7, 60438 Frankfurt/Main, Germany

## Abstract

The title compound, C_14_H_9_Cl_3_N_2_OS, has bond lengths and angles which are quite typical for thio­urea compounds of this class. The mol­ecule exists in the solid state in its thione form with typical thio­urea C=S and C=O bond lengths, as well as shortened C—N bonds. An intra­molecular N—H⋯O hydrogen bond stabilizes the mol­ecular conformation. Inter­molecular N—H⋯S hydrogen bonds link the mol­ecules to form centrosymmetric dimers.

## Related literature

For thio­urea derivatives with biological activities, see: Baily *et al.* (1996 ▶); Koch (2001 ▶); Maryanoff *et al.* (1986 ▶); Namgun *et al.* (2001 ▶); Patil & Chedekel (1984 ▶); Upadlgaya & Srivastava (1982 ▶); Wegner *et al.* (1986 ▶); Krishnamurthy *et al.* (1999 ▶). For related structures, see: Khawar Rauf *et al.* (2006*a* ▶,*b* ▶,*c* ▶, 2007 ▶). For standard bond-length data, see: Allen (2002 ▶).
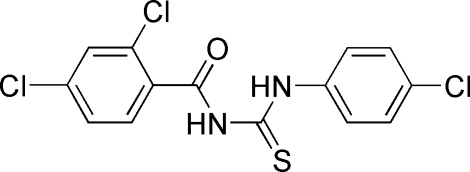


## Experimental

### 

#### Crystal data

C_14_H_9_Cl_3_N_2_OS
*M* *_r_* = 359.65
Triclinic, 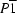

*a* = 5.9674 (6) Å
*b* = 9.6577 (9) Å
*c* = 13.9585 (13) Å
α = 92.919 (6)°
β = 98.005 (7)°
γ = 101.330 (8)°
*V* = 778.54 (13) Å^3^
*Z* = 2
Mo *K*α radiation
μ = 0.72 mm^−1^
*T* = 173 (2) K
0.37 × 0.34 × 0.33 mm

#### Data collection

Stoe IPDS II two-circle diffractometer
Absorption correction: multi-scan (*MULABS*; Spek, 2003 ▶; Blessing, 1995 ▶) *T*_min_ = 0.776, *T*_max_ = 0.797
10758 measured reflections
3418 independent reflections
3154 reflections with *I* > 2σ(*I*)
*R*_int_ = 0.037

#### Refinement

*R*[*F* ^2^ > 2σ(*F* ^2^)] = 0.029
*wR*(*F* ^2^) = 0.078
*S* = 1.02
3418 reflections
199 parameters
H atoms treated by a mixture of independent and constrained refinement
Δρ_max_ = 0.35 e Å^−3^
Δρ_min_ = −0.30 e Å^−3^

### 

Data collection: *X-AREA* (Stoe & Cie, 2001 ▶); cell refinement: *X-AREA*; data reduction: *X-AREA*; program(s) used to solve structure: *SHELXS97* (Sheldrick, 2008 ▶); program(s) used to refine structure: *SHELXL97* (Sheldrick, 2008 ▶); molecular graphics: *XP* in *SHELXTL-Plus* (Sheldrick, 2008 ▶); software used to prepare material for publication: *SHELXL97*.

## Supplementary Material

Crystal structure: contains datablocks I, global. DOI: 10.1107/S160053680900004X/rk2121sup1.cif

Structure factors: contains datablocks I. DOI: 10.1107/S160053680900004X/rk2121Isup2.hkl

Additional supplementary materials:  crystallographic information; 3D view; checkCIF report

## Figures and Tables

**Table 1:** Hydrogen-bond geometry (Å, °)

*D*—H⋯*A*	*D*—H	H⋯*A*	*D*⋯*A*	*D*—H⋯*A*
N1—H1⋯S1^i^	0.84 (2)	2.71 (2)	3.4273 (12)	144.3 (16)
N2—H2⋯O1	0.81 (2)	2.06 (2)	2.7098 (16)	136.4 (19)

Symmetry code: (i) 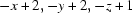.

**Fig. 1.**
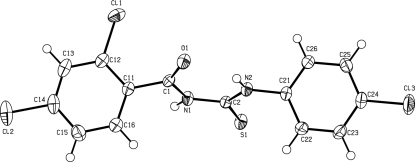
Molecular structure of the title compound showing atom numbering scheme. Displacement ellipsoids are drawn at the 50% probability level. H atoms are drawn as a small spheres of arbitrary radius.

**Fig. 2.**
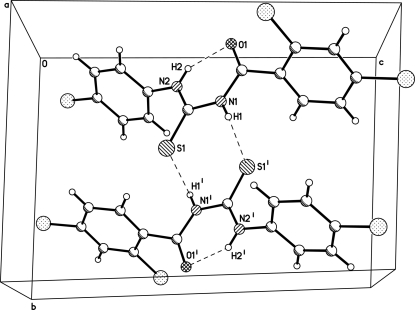
Packing diagram of the title compound with view onto the bc plane. Hydrogen bonds are shown as dashed lines.

## supplementary crystallographic information

### Comment

*N*-substituted and *N*,*N'*-disubstituted thiourea derivatives are very useful starting materials for the synthesis of a wide range of aliphatic macromolecular and heterocyclic compounds. Benzothiazoles have been prepared from arylthioureas in the presence of bromine (Patil & Chedekel, 1984) and condensation of thiourea with α-halocarbonyl compounds form 2-aminothiazoles (Baily *et al.*, 1996). The 2-methyl-aminothiazolines have been synthesized by cyclization of *N*-(2-hydroxyethyl)-*N'*-methylthioureas (Namgun *et al.*, 2001). Thioureas are efficient guanylating agents (Maryanoff *et al.*, 1986). The *N*,*N*-dialkyl-*N*-aroylthioureas have been effectively used for the extraction of Ni, Pd and Pt metals (Koch, 2001). Aliphatic and acylthioureas are well known for their fungicidal, antiviral, pesticidal and plant-growth regulating activities (Upadlgaya & Srivastava, 1982; Wegner *et al.*, 1986). Symmetrical and unsymmetrical thioureas have shown antifungal activity against the plant pathogens *Pyricularia oryzae* and *Drechslera oryzae* (Krishnamurthy *et al.*, 1999). We are interested in the synthesis of these thioureas as intermediates in the synthesis of novel guanidines and heterocyclic compounds for the systematic study of bioactivity and complexation behaviour and we present here the crystal structure of the title compound. The title compound (Fig. 1) shows the typical thiourea C═S and C═O double bonds as well as shortened C—N bond lengths. The thiocarbonyl and carbonyl groups are almost coplanar, as reflected by the torsion angles C2—N1—C1—O1 = 9.0 (2)° and N2—C2—N1—C1 =  5.5 (2)°. This is associated with the expected typical thiourea intramolecular N—H···O hydrogen bond (Table). The dihedral angle formed by the two benzene ring planes is 9.35 (9)°. Bond lengths and angles can be regarded as typical for *N*,*N'*-disubstituted thiourea compounds as found in the Cambridge Structural Database ver. 5.28 (Allen, 2002) and Khawar Rauf *et al.*, 2006*a*,*b*,*c*, 2007. Intermolecular N—H···S hydrogen bonds (Table, Fig. 2), link the molecules to dimers. The Cl atoms are not involved in any type of hydrogen bonds.

### Experimental

Freshly prepared 2,4-dichlorobenzoyl isothiocyanate (2.3 g, 10 mmol) was stirred in acetone (40 ml) for 20 min. Neat 4-chloroaniline (1.3 g, 10 mmol) was then added and the resulting mixture was stirred for 1.5 h. The reaction mixture was then poured into acidified (pH 4) water and stirred well. The solid product was separated and washed with deionized water and purified by recrystallization from methanol–1,1-dichloromethane (1:10 *v*/*v*) to give fine crystals of title compound, with an overall yield of 90%. Full spectroscopic and physical characterization will be reported elsewhere.

### Refinement

H atoms were located in a difference map, but those bonded to C were refined with fixed individual displacement parameters *U*_iso_(H) = 1.2*U*_eq_(C) using a riding model with C—H = 0.95 Å. The H atoms bonded to N were refined freely.

### Crystal data

**Table:**

C_14_H_9_Cl_3_N_2_OS	*Z* = 2
*M**_r_* = 359.65	*F*(000) = 364
Triclinic, *P*1	*D*_x_ = 1.534 Mg m^−^^3^
Hall symbol: -P 1	Mo *K*α radiation, λ = 0.71073 Å
*a* = 5.9674 (6) Å	Cell parameters from 8758 reflections
*b* = 9.6577 (9) Å	θ = 3.7–27.1°
*c* = 13.9585 (13) Å	µ = 0.72 mm^−^^1^
α = 92.919 (6)°	*T* = 173 K
β = 98.005 (7)°	Block, colourless
γ = 101.330 (8)°	0.37 × 0.34 × 0.33 mm
*V* = 778.54 (13) Å^3^	

### Data collection

**Table:**

Stoe IPDS II two-circle diffractometer	3418 independent reflections
Radiation source: fine-focus sealed tube	3154 reflections with *I* > 2σ(*I*)
graphite	*R*_int_ = 0.037
ω scans	θ_max_ = 27.1°, θ_min_ = 3.5°
Absorption correction: multi-scan (*MULABS*; Spek, 2003; Blessing, 1995)	*h* = −7→7
*T*_min_ = 0.776, *T*_max_ = 0.797	*k* = −12→12
10758 measured reflections	*l* = −16→17

### Refinement

**Table:**

Refinement on *F*^2^	Secondary atom site location: difference Fourier map
Least-squares matrix: full	Hydrogen site location: inferred from neighbouring sites
*R*[*F*^2^ > 2σ(*F*^2^)] = 0.029	H atoms treated by a mixture of independent and constrained refinement
*wR*(*F*^2^) = 0.078	*w* = 1/[σ^2^(*F*_o_^2^) + (0.0411*P*)^2^ + 0.3093*P*] where *P* = (*F*_o_^2^ + 2*F*_c_^2^)/3
*S* = 1.02	(Δ/σ)_max_ = 0.001
3418 reflections	Δρ_max_ = 0.35 e Å^−^^3^
199 parameters	Δρ_min_ = −0.30 e Å^−^^3^
0 restraints	Extinction correction: *SHELXL97* (Sheldrick, 2008), Fc^*^=kFc[1+0.001xFc^2^λ^3^/sin(2θ)]^-1/4^
Primary atom site location: structure-invariant direct methods	Extinction coefficient: 0.032 (3)

### Special details

**Table:**

Geometry. All s.u.'s (except the s.u. in the dihedral angle between two l.s. planes) are estimated using the full covariance matrix. The cell s.u.'s are taken into account individually in the estimation of s.u.'s in distances, angles and torsion angles; correlations between s.u.'s in cell parameters are only used when they are defined by crystal symmetry. An approximate (isotropic) treatment of cell s.u.'s is used for estimating s.u.'s involving l.s. planes.
Refinement. Refinement of *F*^2^ against ALL reflections. The weighted *R*–factor *wR* and goodness of fit *S* are based on *F*^2^, conventional *R*–factors *R* are based on *F*, with *F* set to zero for negative *F*^2^. The threshold expression of *F*^2^ > σ(*F*^2^) is used only for calculating *R*–factors(gt) *etc*. and is not relevant to the choice of reflections for refinement. *R*–factors based on *F*^2^ are statistically about twice as large as those based on *F* and *R*–factors based on ALL data will be even larger.

### Fractional atomic coordinates and isotropic or equivalent isotropic displacement parameters (Å^2^)

**Table:**

	*x*	*y*	*z*	*U*_iso_*/*U*_eq_	
S1	0.67492 (6)	0.98619 (3)	0.38939 (2)	0.02427 (11)	
Cl1	1.21954 (7)	0.58845 (5)	0.63698 (3)	0.03934 (12)	
Cl2	1.28238 (9)	0.84274 (6)	0.99473 (3)	0.05304 (15)	
Cl3	−0.25621 (7)	0.63336 (6)	0.06553 (3)	0.04618 (14)	
O1	0.69489 (18)	0.58953 (10)	0.55419 (8)	0.0271 (2)	
N1	0.7744 (2)	0.82647 (12)	0.53113 (8)	0.0217 (2)	
H1	0.870 (3)	0.901 (2)	0.5518 (14)	0.031 (5)*	
N2	0.48353 (19)	0.71427 (12)	0.40789 (8)	0.0214 (2)	
H2	0.488 (3)	0.644 (2)	0.4369 (15)	0.036 (5)*	
C1	0.7879 (2)	0.71114 (13)	0.58426 (10)	0.0198 (2)	
C2	0.6359 (2)	0.83297 (13)	0.44250 (9)	0.0194 (2)	
C11	0.9208 (2)	0.74977 (13)	0.68462 (10)	0.0211 (3)	
C12	1.1140 (2)	0.69336 (14)	0.71709 (11)	0.0253 (3)	
C13	1.2283 (3)	0.72390 (16)	0.81191 (11)	0.0319 (3)	
H13	1.3614	0.6870	0.8331	0.038*	
C14	1.1437 (3)	0.80951 (17)	0.87478 (11)	0.0332 (3)	
C15	0.9539 (3)	0.86812 (18)	0.84524 (12)	0.0357 (3)	
H15	0.8995	0.9269	0.8895	0.043*	
C16	0.8441 (3)	0.83931 (16)	0.74932 (11)	0.0294 (3)	
H16	0.7162	0.8808	0.7277	0.035*	
C21	0.3125 (2)	0.70259 (13)	0.32315 (9)	0.0202 (3)	
C22	0.1340 (2)	0.77684 (15)	0.32197 (11)	0.0266 (3)	
H22	0.1311	0.8405	0.3757	0.032*	
C23	−0.0402 (2)	0.75754 (16)	0.24188 (11)	0.0290 (3)	
H23	−0.1616	0.8083	0.2401	0.035*	
C24	−0.0327 (2)	0.66248 (16)	0.16468 (10)	0.0274 (3)	
C25	0.1444 (3)	0.58874 (16)	0.16452 (10)	0.0288 (3)	
H25	0.1471	0.5253	0.1106	0.035*	
C26	0.3185 (2)	0.60921 (14)	0.24475 (10)	0.0246 (3)	
H26	0.4411	0.5594	0.2459	0.030*	

### Atomic displacement parameters (Å^2^)

**Table:**

	*U*^11^	*U*^22^	*U*^33^	*U*^12^	*U*^13^	*U*^23^
S1	0.02479 (18)	0.01930 (16)	0.02596 (18)	−0.00011 (12)	−0.00124 (13)	0.00791 (12)
Cl1	0.0333 (2)	0.0460 (2)	0.0449 (2)	0.02251 (17)	0.00655 (16)	0.00362 (17)
Cl2	0.0557 (3)	0.0652 (3)	0.0274 (2)	−0.0014 (2)	−0.01264 (18)	0.00720 (19)
Cl3	0.0303 (2)	0.0761 (3)	0.0284 (2)	0.01286 (19)	−0.00926 (15)	−0.00085 (19)
O1	0.0275 (5)	0.0176 (4)	0.0327 (5)	0.0013 (4)	−0.0038 (4)	0.0041 (4)
N1	0.0221 (5)	0.0163 (5)	0.0228 (6)	−0.0012 (4)	−0.0029 (4)	0.0031 (4)
N2	0.0222 (5)	0.0169 (5)	0.0227 (5)	0.0015 (4)	−0.0022 (4)	0.0040 (4)
C1	0.0160 (6)	0.0204 (6)	0.0232 (6)	0.0038 (4)	0.0026 (5)	0.0045 (5)
C2	0.0186 (6)	0.0194 (6)	0.0199 (6)	0.0037 (4)	0.0015 (5)	0.0021 (4)
C11	0.0196 (6)	0.0194 (6)	0.0231 (6)	0.0015 (4)	0.0014 (5)	0.0058 (5)
C12	0.0209 (6)	0.0251 (6)	0.0299 (7)	0.0046 (5)	0.0026 (5)	0.0080 (5)
C13	0.0233 (7)	0.0353 (8)	0.0351 (8)	0.0031 (6)	−0.0031 (6)	0.0145 (6)
C14	0.0335 (8)	0.0365 (8)	0.0232 (7)	−0.0036 (6)	−0.0039 (6)	0.0085 (6)
C15	0.0420 (9)	0.0382 (8)	0.0263 (7)	0.0090 (7)	0.0032 (6)	0.0003 (6)
C16	0.0299 (7)	0.0315 (7)	0.0277 (7)	0.0105 (6)	0.0013 (6)	0.0028 (6)
C21	0.0189 (6)	0.0185 (6)	0.0209 (6)	0.0002 (4)	−0.0002 (5)	0.0045 (5)
C22	0.0251 (7)	0.0249 (6)	0.0289 (7)	0.0066 (5)	0.0006 (5)	−0.0020 (5)
C23	0.0217 (6)	0.0325 (7)	0.0330 (7)	0.0092 (5)	−0.0001 (6)	0.0028 (6)
C24	0.0216 (6)	0.0364 (7)	0.0217 (6)	0.0025 (5)	−0.0014 (5)	0.0051 (5)
C25	0.0298 (7)	0.0346 (7)	0.0211 (6)	0.0066 (6)	0.0022 (5)	−0.0014 (5)
C26	0.0232 (6)	0.0265 (6)	0.0246 (7)	0.0073 (5)	0.0020 (5)	0.0029 (5)

### Geometric parameters (Å, °)

**Table:**

S1—C2	1.6786 (13)	C13—H13	0.9500
Cl1—C12	1.7336 (15)	C14—C15	1.385 (2)
Cl2—C14	1.7454 (16)	C15—C16	1.395 (2)
Cl3—C24	1.7529 (14)	C15—H15	0.9500
O1—C1	1.2217 (16)	C16—H16	0.9500
N1—C1	1.3784 (16)	C21—C26	1.3912 (19)
N1—C2	1.4003 (17)	C21—C22	1.3947 (19)
N1—H1	0.84 (2)	C22—C23	1.394 (2)
N2—C2	1.3365 (17)	C22—H22	0.9500
N2—C21	1.4348 (16)	C23—C24	1.391 (2)
N2—H2	0.81 (2)	C23—H23	0.9500
C1—C11	1.5004 (18)	C24—C25	1.385 (2)
C11—C12	1.3993 (19)	C25—C26	1.395 (2)
C11—C16	1.401 (2)	C25—H25	0.9500
C12—C13	1.391 (2)	C26—H26	0.9500
C13—C14	1.387 (2)		
			
C1—N1—C2	128.79 (11)	C14—C15—C16	118.83 (15)
C1—N1—H1	115.9 (13)	C14—C15—H15	120.6
C2—N1—H1	115.1 (13)	C16—C15—H15	120.6
C2—N2—C21	124.59 (11)	C15—C16—C11	120.59 (14)
C2—N2—H2	117.6 (14)	C15—C16—H16	119.7
C21—N2—H2	117.8 (14)	C11—C16—H16	119.7
O1—C1—N1	123.60 (12)	C26—C21—C22	120.47 (12)
O1—C1—C11	122.92 (12)	C26—C21—N2	119.04 (12)
N1—C1—C11	113.44 (11)	C22—C21—N2	120.35 (12)
N2—C2—N1	115.97 (11)	C23—C22—C21	119.97 (13)
N2—C2—S1	125.98 (10)	C23—C22—H22	120.0
N1—C2—S1	118.06 (9)	C21—C22—H22	120.0
C12—C11—C16	118.82 (13)	C24—C23—C22	118.73 (13)
C12—C11—C1	121.64 (12)	C24—C23—H23	120.6
C16—C11—C1	119.48 (12)	C22—C23—H23	120.6
C13—C12—C11	121.15 (14)	C25—C24—C23	121.94 (13)
C13—C12—Cl1	119.15 (11)	C25—C24—Cl3	119.11 (11)
C11—C12—Cl1	119.66 (11)	C23—C24—Cl3	118.95 (11)
C14—C13—C12	118.49 (14)	C24—C25—C26	118.95 (13)
C14—C13—H13	120.8	C24—C25—H25	120.5
C12—C13—H13	120.8	C26—C25—H25	120.5
C15—C14—C13	122.07 (14)	C21—C26—C25	119.93 (13)
C15—C14—Cl2	119.67 (13)	C21—C26—H26	120.0
C13—C14—Cl2	118.26 (12)	C25—C26—H26	120.0
			
C2—N1—C1—O1	9.0 (2)	C13—C14—C15—C16	0.3 (2)
C2—N1—C1—C11	−168.72 (12)	Cl2—C14—C15—C16	−179.43 (12)
C21—N2—C2—N1	174.17 (12)	C14—C15—C16—C11	1.6 (2)
C21—N2—C2—S1	−6.02 (19)	C12—C11—C16—C15	−2.0 (2)
C1—N1—C2—N2	5.5 (2)	C1—C11—C16—C15	175.05 (13)
C1—N1—C2—S1	−174.29 (11)	C2—N2—C21—C26	118.68 (15)
O1—C1—C11—C12	59.43 (18)	C2—N2—C21—C22	−65.50 (18)
N1—C1—C11—C12	−122.82 (13)	C26—C21—C22—C23	0.1 (2)
O1—C1—C11—C16	−117.54 (15)	N2—C21—C22—C23	−175.68 (13)
N1—C1—C11—C16	60.22 (16)	C21—C22—C23—C24	0.7 (2)
C16—C11—C12—C13	0.5 (2)	C22—C23—C24—C25	−1.1 (2)
C1—C11—C12—C13	−176.50 (12)	C22—C23—C24—Cl3	177.91 (11)
C16—C11—C12—Cl1	−177.26 (11)	C23—C24—C25—C26	0.8 (2)
C1—C11—C12—Cl1	5.75 (17)	Cl3—C24—C25—C26	−178.21 (11)
C11—C12—C13—C14	1.4 (2)	C22—C21—C26—C25	−0.4 (2)
Cl1—C12—C13—C14	179.14 (11)	N2—C21—C26—C25	175.42 (12)
C12—C13—C14—C15	−1.8 (2)	C24—C25—C26—C21	−0.1 (2)
C12—C13—C14—Cl2	177.96 (11)		

### Hydrogen-bond geometry (Å, °)

**Table:**

*D*—H···*A*	*D*—H	H···*A*	*D*···*A*	*D*—H···*A*
N1—H1···S1^i^	0.84 (2)	2.71 (2)	3.4273 (12)	144.3 (16)
N2—H2···O1	0.81 (2)	2.06 (2)	2.7098 (16)	136.4 (19)

Symmetry codes: (i) −*x*+2, −*y*+2, −*z*+1.
